# The Reciprocal Relationship Between Handwriting Fluency and Spelling Accuracy in Chinese: A Longitudinal Study

**DOI:** 10.3389/fpsyg.2020.00620

**Published:** 2020-04-15

**Authors:** Yuan Ding, Liping Li, Xinchun Wu

**Affiliations:** ^1^School of Education Science, Collaborative Innovation Center for Fundamental Education Quality Enhancement of Shanxi Province, Experimental Teaching Center of Psychology and Cognitive Behavior, Shanxi Normal University, Linfen, China; ^2^Research Center of Children's Reading and Learning, Beijing Key Laboratory of Applied Experimental Psychology, Faculty of Psychology, Beijing Normal University, Beijing, China

**Keywords:** reciprocal relationship, handwriting fluency, spelling accuracy, Chinese children, longitudinal study

## Abstract

Mastering transcription skills is an important goal in the development of children's written language abilities, and handwriting fluency and spelling accuracy are crucial indicators of transcription ability. The current study was a two-year longitudinal study to investigate the reciprocal relationship of handwriting fluency and spelling accuracy. Participants included 123 students living in mainland China, who were tracked from third to fifth grade, and were administered a comprehensive battery of tests including assessments for non-verbal intelligence, phonological awareness, rapid automatized naming, and copying and dictation of Chinese characters. The results showed that: (1) previous handwriting fluency predicted subsequent spelling accuracy; and (2) previous spelling accuracy predicted subsequent handwriting fluency. These findings indicated there is a bidirectional relationship between handwriting fluency and spelling accuracy in Chinese. This implies spelling accuracy should not be unilaterally emphasized when teaching children new vocabulary, but attention should also be given to the cultivation of handwriting fluency in daily pedagogical practice.

## Introduction

Transcription is the ability to transform linguistic representations in working memory into written texts, and incorporates both handwriting and spelling skills (Berninger, 1999). According to the simple view of writing (Berninger et al., 2002; Bisschop et al., 2016), transcription, as one of its vital related skills, supports the complex writing processes performed within working memory. However, while the simple view of writing emphasizes the importance of transcription skills, it fails to indicate the relationship between handwriting and spelling, the two essential skills needed for transcription. Compared to the considerable amount of research regarding transcription and writing (Limpo and Alves, 2013; Kent et al., 2014), the interaction between handwriting and spelling skills have not attracted much attention in the literacy acquisition process (Medwell and Wray, 2010). The widespread use of mobile phones and computers makes individuals gradually become used to typing over writing by hand, which seriously weakens writing skills and results in having problems writing characters (Hu, 2019). Nevertheless, both handwriting and spelling skills are vital for individuals to learn, especially children. It is estimated that 30–60% of a child's school day is spent performing written work (Marr et al., 2001). Children should be able to master certain levels of handwriting and spelling skills so they can adeptly use them as tools to improve their learning in school (Limpo et al., 2017). Thus, fluent handwriting and accurate spelling can help to minimize restrictions on students' writing and facilitate their acquisition of basic writing skills (Graham and Santangelo, 2014), even enabling students to achieve maximum success in the classroom throughout their academic careers (Eames and Loewenthal, 1990; Graham et al., 2000).

Handwriting is a complex activity requiring an intricate blend of cognitive, kinesthetic, and perceptual-motor components (Rosenblum et al., 2003), and it is influenced by children's visual motor integration, fine motor dexterity, and other skills. Each of these is considered the foundational skill required for adequate handwriting (Maeland, 1992). The more automatized this process of integration is, the more cognitive resources are available, and handwriting becomes smoother under a lower cognitive load (Bourdin and Fayol, 1994). Hence, whether English (Berninger et al., 1992; Lambert et al., 2011) or Chinese studies (McBride-Chang et al., 2011; Yeung et al., 2016), handwriting skills, especially handwriting fluency, has always been a major focus in research on children's writing development. In the handwriting practice, children are required to write as fast as possible, and their handwriting fluency are assessed by counting the number of correct letters written during the task (Berninger et al., 1992). During this process, children who want to write quickly need to build and strengthen their visual-spatial representation skills and orthographic characterization of words. The integration of this ability is directly related to the level of automation, thus affecting the speed of children's output. Moreover, the automatization of this process means that children have high levels of handwriting fluency (Santangelo and Graham, 2016).

Furthermore, spelling is also an important component of transcription (Berninger et al., 1992). Spelling refers to the ability to recognize, recall, and reconstruct the correct order of letters for a word in spoken or written form (Graham and Miller, 1979). Children are often required to understand and apply phonetic and morphological rules to establish orthographic representations of words (Critten et al., 2016). Moreover, spelling is a complex cognitive process that emphasizes accuracy. Spelling accuracy is a core indicator of children's spelling abilities, and dictation has been conventionally adopted to test spelling accuracy in alphabetic languages (Lam and McBride, 2018) and morphosyllabic languages (Li et al., 2017)]. In the process of dictation, when children hear a spoken word, they need to spell out this word, with the help of grapheme-phoneme correspondence and complex orthographic rules (Brown and Ellis, 1994). Correct spelling in dictation indicates that a child has a firm grasp of words and good spelling accuracy (Morris, 1983).

Understanding the bidirectional relationship between handwriting fluency and spelling accuracy is significant for educational researchers when attempting to improve these skills. In contrast to the substantial amount of research examining the direct effects of handwriting fluency and spelling accuracy on writing and reading performance (Graham et al., 1997; Graham and Harris, 2000; Limpo and Alves, 2013), there are only a handful of studies investigating the underlying mechanism of the relationship between handwriting and spelling. By analyzing the influencing factors of spelling accuracy in children in the third grade, researchers (Cheng-Lai et al., 2013) concluded that the degree of difficulty in performing Chinese word dictation co-varies with handwriting fluency. However, from a developmental perspective, the bidirectional relationship between handwriting fluency and spelling accuracy remains unclear. Given that cognitive skills, such as visual-orthographic coding, required for handwriting fluency overlap with spelling skills (McBride-Chang et al., 2011), it is reasonable to assume that there is a mutually reinforcing relationship between handwriting fluency and spelling accuracy.

### The Role of Handwriting Fluency in Spelling Accuracy

Automatic information processing (LaBerge and Samuels, 1974) refers to the theory that repetition can gradually allow the brain to use fewer resources to focus on details, making a behavior easier to perform. Therefore, it requires repeated practice to improve the automatization of information processing and task quality. This theory seems to apply equally to handwriting. The perceptual and kinesthetic aspects of handwriting are integrated with the language network in the brain, and this connection can become increasingly close, even automatic, which frees up more attention resources and further improves spelling accuracy. Hence, handwriting fluency may be potentially crucial for children's later development of spelling skills.

In a prior study about Chinese language (McBride-Chang et al., 2011), Hong Kong children in the third and fourth grades, with and without dyslexia, were administrated tasks of copying unfamiliar prints and dictation. The results showed that the correlation coefficient between handwriting fluency and spelling accuracy was between 0.37 and 0.58, and handwriting fluency explained 3% of the unique variance in spelling accuracy. Consistent with findings from McBride-Chang et al. (2011), Lam and McBride (2018) surveyed 141 kindergartners in Hong Kong schools to explore the important role of handwriting skills in how children learn to spell Chinese words, and found that handwriting altogether significantly explained 10% of the variance in spelling accuracy, even after statistically controlling for the effects of age, non-verbal IQ, vocabulary knowledge, morphological awareness, orthographic awareness, and phonological awareness. Similar results were also seen in studies by Bosga-Stork et al. (2016) and Afonso et al. (2018), however, they further found that the effect only lasted until the third grade and disappeared in upper grades. Thus, they believed handwriting to be independent of spelling by the third grade. On the contrary, practical teaching experience showed that a vast majority of primary school teachers generally train students' mastery of words by means of handwriting practice, and teachers believed that for students who are fluent in handwriting, they often spell more accurate words in spelling assignment (Graham et al., 2008). As a result, it is unclear whether handwriting and spelling develop independently in upper grade levels. Additionally, the above studies generally adopted the cross-sectional design to explore correlations between variables, so it's hard to examine whether the previous variable would affect subsequent variable. Longitudinal studies may be better able to examine causal relationships between variables over time, thus, whether previous handwriting fluency has an impact on later spelling accuracy in upper primary school grades needs to be studied further.

### The Role of Spelling Accuracy in Handwriting Fluency

The lexical quality hypothesis (Perfetti and Hart, 2002) posits that the quality of lexical representation depends on three dimensions: phonology, orthography, and semantics. All three are indispensable, as without them, the overall effect of lexical representation would be impacted, such as the accuracy and efficiency of lexical recognition. Furthermore, proficiency in literacy skills depends on the utilization frequency of an individual's high-quality lexical representations. In other words, once an individual has a good grasp of the depth of the vocabulary, the refinement of lexical information processing can effectively form abundant network connections in the brain. As a result, automatic lexical representation is gradually formed, and, accordingly, the retrieval of vocabulary is more efficient. Therefore, investigating spelling accuracy could help increase understanding of the development of children's handwriting fluency.

There have been studies on the effects of spelling accuracy on handwriting fluency; however, most have been conducted using children with dyslexia, and relatively few examined children without dyslexia. As evidenced by an empirical study analyzing the spelling errors of 10-year-old children with dyslexia, Martlew (1992) discovered that spelling accuracy affects handwriting fluency and pause times. Similarly, Sumner et al. (2014) investigated children with dyslexia, and the findings showed that spelling accuracy significantly accounted for 53% of the variances in their handwriting fluency, which indicated that poor spelling in children with dyslexia could limit the rate of handwriting production. The effect is particularly pronounced in children with dyslexia; however, whether it can be extended to children without dyslexia remains unclear. Abbott et al. (2010) followed non-dyslexic English-speaking children in the first through seventh grades, and found that the longitudinal path from spelling accuracy to handwriting fluency was significant across adjacent grades, from fourth to fifth. After all, the above studies are based on alphabetic languages, and the clues of correspondence between letters and phonemes can be used (Jiang, 2001). However, in Chinese, there are many homophones, grapheme-phoneme correspondence is arbitrary, and phonetic clues are unreliable (Shu et al., 2003). Given the different characteristics between Chinese characters and the English alphabet, it is unclear how much present spelling accuracy would affect later handwriting fluency for non-dyslexic Chinese-speaking children.

### Chinese Writing

Different from the English alphabet, which is composed of 26 letters, Chinese writing uses square script and morphosyllabic characters. Chinese characters can be broken down into radicals and strokes. Although about 80% of the phonetic radicals in modern Chinese characters provide clues for their pronunciation (Shu et al., 2003), the grapheme-phoneme correspondence can be arbitrary. Therefore, retrieving the correct character from short-term memory can be difficult for children. Furthermore, Chinese characters have rich visual-spatial properties (Kao et al., 2004). Through the mapping of Chinese characters' cognitive images, effective handwriting practice could help children become familiar with the visual-spatial properties of the characters and facilitate children's orthographic awareness and establishment of long-term motor memory (Tan et al., 2005). Thus, there is an alternative possibility that children may develop spelling accuracy through handwriting fluency. On the other hand, familiar with the strokes and radicals of Chinese characters may also help children to maintain the continuous output of character, which in turn promotes the development of handwriting fluency. Therefore, there may be a reciprocal relationship between handwriting fluency and spelling accuracy in Chinese.

### This Study

Previous research has provided preliminary evidence for a potential relationship between handwriting fluency and spelling accuracy. The primary aim of the current longitudinal study (two-year follow up) was to evaluate the mutual causality between handwriting fluency and spelling accuracy in upper primary school grades. On the basis of the above-mentioned theoretical concepts and empirical results, we hypothesized that a bidirectional relationship exists between handwriting fluency and spelling accuracy. Previous studies found that non-verbal IQ is a powerful predictor of handwriting and spelling skills (Sampson et al., 2003). Further, phonological awareness and rapid automatized naming also play important roles in word recognition and production (Savage et al., 2008; Alloway and Alloway, 2010). Therefore, non-verbal IQ, phonological awareness, and rapid automatized naming were the control variables in the current study.

## Methods

### Participants

The study was approved by the Research Ethics Committee of Beijing Normal University. The sample consisted of 136 children, who are selected by cluster sampling from two primary schools in the Shanxi province in mainland China. Thirteen participants (9% attrition rate) were eliminated due to transferring to other schools. The final sample size was 123 children (62 boys and 61 girls), with no significant differences in intelligence [*t*_(123)_ = −1.24, *p* = 0.22] or gender [χ ^2^(1) = 1.67, *p* = 0.25] between children included in the sample and those who had withdrawn from the study. School principals, classroom teachers, and parents supported our study, were informed of its purpose, and provided written informed consent.

### Measures

#### Handwriting Fluency

We adopted a prior digit copying fluency task (Yan et al., 2012) and a sentence copying fluency task (Guan et al., 2013) to assess children's handwriting fluency. In the digit copying fluency task, children were required to copy in 1 min, repeatedly and as quickly as possible, a string of digits line by line (e.g., 一二三四五六七八九十; one, two, three, four, five, six, seven, eight, nine, and ten). The test was scored by the total number of words copied. The test-retest reliability coefficient for the fourth and fifth grade tests was 0.78. In the sentence copying fluency task, children were asked to copy a sentence (e.g., 敏捷的棕狐狸跳越懒狗; the quick brown fox jumps over the lazy dog). This sentence uses complicated words which contained almost the full range of single strokes (see sample items in Appendix). Before the test, children were required to be familiar with the sentence, so as to ensure the children knew its meaning. Thus, the influence of syntactic skills on children's copying speed could be reduced as much as possible. For the task, children were asked to repeatedly copy the sentence as quickly as possible within 1 min. The total score on the test was the correct number of words copied in sequence. The test-retest reliability coefficient of this task for the fourth and fifth grade tests was 0.71. The participants were then given the same test in the fourth and fifth grades.

#### Spelling Accuracy

Regarding Chinese character dictation tasks (Li et al., 2017), easy-items and difficult-items dictation tasks were used to examine spelling accuracy. Children were asked to dictate a target word in disyllabic words, such as “读” /du2/ [read] in “读书” /du2 shu1/ [read books]. Twelve easy items were selected from Chinese textbooks for their current grade level, and twelve difficult items were selected from Chinese textbooks of participants' next grade level. Compared with the terms of current grade level, the terms of next grade level is more difficult for participants. The 12 difficult items were the low-frequency words, and the frequency is about 0.001% (Modern Chinese Frequency Dictionary, 1986). The participants were asked to write down each target word, which was repeated twice on the recording, first in isolation and then embedded in a two-character word (sample items appear in Appendix). Children were encouraged to write as many words as possible. Correctly writing a word was scored as 1 point. The participants were given different dictation tasks in the fourth and fifth grades. Cronbach's α coefficients for easy items of the fourth and fifth grade tests were 0.70 and 0.74, respectively. And Cronbach's α coefficients for difficult items of the fourth and fifth grade tests were 0.72 and 0.77, respectively.

#### Rapid Automatized Naming

Rapid digit naming task contained five numbers, 1, 3, 4, 5, and 8, presented in a 5 × 5 matrix on a single sheet of paper, and was used to determine child's ability to quickly pronounce the numbers. There was only 1 trial, children were required to read the digit matrix aloud twice, as quickly and accurately as possible each time. The average score of the two tests was used as the final score. The experimenter timed the tests with a stopwatch, which was accurate to 0.01 s. The test-retest reliability coefficient for this task was 0.83.

#### Phonological Awareness

The phoneme deletion task (Shu et al., 2006) was used to determine children's perceptual and operational capability of phonology. For this task, the children were asked to produce a new syllable by taking away the target phoneme from a monosyllabic Chinese word, including deletion of the initial, middle, and last phonemes of target syllables, respectively. This task comprised 6 practice items and 12 test items. A correct response received 1 point, for 12 points possible in total. Cronbach's α coefficient for this task was 0.74.

#### Nonverbal IQ

Using the standardized Chinese version of Raven's Progressive Matrices (Zhang and Wang, 1985), children were asked to select the most appropriate choice from six to eight choices to complete the target pattern. There were 60 items, and Cronbach's α coefficient for this task was 0.93.

### Procedure

As part of longitudinal research on the literacy development of Chinese children, the current study evaluated children's handwriting fluency and spelling accuracy development over 2 years. The experimenter, with the assistance of a class teacher, administered Raven's Progressive Matrices and the Chinese character dictation tasks to participants. These tasks were taken by classes as a unit (45 students), and three classes were tested. Other tests were carried out individually, including the phoneme deletion task, rapid automatized naming tasks, digit copying fluency task and sentence copying fluency task. The duration of each session was 40 min in total. The whole study consisted of four time points. Table 1 shows the tests and procedures. Control variables were measured at the first two time points, i.e., participants' non-verbal IQs in the autumn semester of first grade (T0), phonological awareness and rapid automatized naming abilities in the autumn semester of third grade (T1). The handwriting fluency and spelling accuracy tests were conducted in the participants' autumn semester of fourth grade (T2) and re-assessed in fifth grade (T3).

**Table 1:** Tests and procedures.

**Test time**	**Variables**	**Task**
First grade (T0)	Intelligence	Raven's progressive matrices
Third grade (T1)	Phonological awareness	Phoneme deletion task
	Rapid automatized naming	Rapid automatized naming tasks
Fourth grade (T2)	Handwriting fluency	Digit copying fluency task
		Sentence copying fluency task
	Spelling accuracy	Easy items dictation task
		Difficult items dictation task
Fifth grade (T3)	Handwriting fluency	Digit copying fluency task
		Sentence copying fluency task
	Spelling accuracy	Easy items dictation task
		Difficult items dictation task

## Results

### Descriptive Results of Each Variable

Table 2 presents means and standard deviations computed for all measurements of this study. Children's handwriting fluency improved with the increase of grade levels (digit copying task: *F*_(1, 122)_ = 68.79, *p* < 0.001; sentence copying task: *F*_(1, 122)_ = 151.66, *p* < 0.001). Due to the different tests used for fourth and fifth grade, the comparison of spelling accuracy could not be carried out.

**Table 2:** Descriptive statistics for all variables at all time points (M ± SD).

	**T0**	**T1**	**T2**	**T3**
IQ	27.40 ± 8.74	–	–	–
PA	–	9.20 ± 2.31	–	–
RAN	–	9.15 ± 2.16	–	–
DC	–	–	49.41 ± 10.18	58.12 ± 10.76
SC	–	–	13.75 ± 2.86	17.07 ± 2.69
EI	–	–	10.25 ± 1.68	9.81 ± 2.29
DI	–	–	7.86 ± 2.65	7.42 ± 2.79

*IQ, Raven's Standard Progressive Matrices; PA, Phonological awareness; RAN, Rapid automatized naming; DC, Digit copying fluency task; SC, Sentence copying fluency task; EI, Easy items; DI, Difficult items; T0, Time 0; T1, Time 1; T2, Time 2; T3, Time 3*.

Table 3 displays correlations between the variables for all time points. The results showed that, rapid automatized naming at T1 had significant correlation with all observed variables about handwriting and spelling at T2 and T3, except for sentence copying at T3. Handwriting fluency at T3 was significantly correlated with spelling accuracy at T3; however, only sentence copying at T2 was significantly correlated with spelling accuracy at T2. Previous handwriting fluency and spelling accuracy at T2 were significantly correlated with later handwriting fluency and spelling accuracy at T3, respectively. Spelling accuracy at T2 was significantly correlated with handwriting fluency at T3; similarly, handwriting fluency at T2 was significantly correlated with spelling accuracy at T3, except for the difficult-items dictation at T3.

**Table 3:** Correlation coefficients for all variables.

	**1**	**2**	**3**	**4**	**5**	**6**	**7**	**8**	**9**	**10**
IQ T0	–									
PA T1	0.13									
RAN T1	−0.02	−0.26^**^								
DC T2	0.03	0.04	−0.24^**^							
SC T2	0.09	0.02	−0.18^*^	0.64^***^						
EI T2	0.04	0.11	−0.25^**^	0.07	0.31^***^					
DI T2	0.20^*^	0.26^**^	−0.34^***^	0.13	0.28^**^	0.70^***^				
DC T3	0.32^***^	0.11	−0.23^*^	0.38^***^	0.38^***^	0.21^*^	0.31^***^			
SC T3	0.22^*^	0.03	−0.14	0.27^**^	0.42^***^	0.19^*^	0.22^*^	0.67^***^		
EI T3	0.25^**^	0.23^*^	−0.23^**^	0.19^*^	0.35^***^	0.66^***^	0.68^***^	0.33^***^	0.20^*^	
DI T3	0.27^**^	0.20^*^	−0.28^**^	0.16	0.27^**^	0.49^***^	0.55^***^	0.40^***^	0.32^***^	0.74^***^

*IQ, Raven's Standard Progressive Matrices; PA, Phonological awareness; RAN, Rapid automatized naming; DC, Digit copying fluency task; SC, Sentence copying fluency task; EI, Easy items dictation task; DI, Difficult items dictation task; T0, Time 0; T1, Time 1; T2, Time 2; T3, Time 3*.

* *p < 0.05*,

** *p < 0.01*,

*** *p < 0.001*.

### Model Analysis

**Figure 1:**
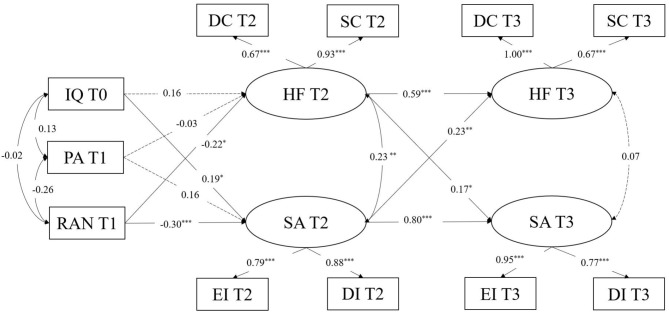
The cross-lagged analysis for handwriting fluency (HF) and spelling accuracy (SA). IQ, Raven's Standard Progressive Matrices; PA, Phonological awareness; RAN, Rapid automatized naming; DC, Digit copying fluency task; SC, Sentence copying fluency task; EI, Easy items dictation task; DI, Difficult items dictation task; T0, Time 0; T1, Time 1; T2, Time 2; T3, Time 3. *p < 0.05, **p < 0.01, ***p < 0.001.

Controlling for the three influencing variables, a two-wave cross-lagged model was used to explore the reciprocal relationship between handwriting fluency and spelling accuracy. A structural equation model was created for data analyses using Amos 22.0 statistical software. Handwriting fluency was a latent variable extracted by two measures (digit copying and sentence copying) as indicators. Spelling accuracy was a latent variable which was extracted by two measures (easy items and difficult items) as indicators. Figure 1 shows the model of the reciprocal relationship between handwriting fluency and spelling accuracy, controlling for non-verbal IQ, phonological awareness, and rapid automatized naming. The model fit index was χ^2^ = 68.51, *df* = 32, root mean square error of approximation (RMSEA) = 0.09, comparative fit index (CFI) = 0.93, incremental fit index (IFI) = 0.93, and Tucker-Lewis index (TLI) = 0.89. According to previous recommendations for good fit indices (Hu and Bentler, 1998), the ratio of χ^2^ to *df* should be smaller than 2; CFI, IFI, and TLI values should be larger than 0.90; and RMSEA values should be smaller than 0.08. In this model, the fit index of TLI was lower and the RMSEA value was larger. According to the modified index model, sentence copying at T2 was correlated with digit copying at T3. When this variable was allowed to correlate with its corresponding variables in the modified model, the fit indices of the modified model were better: χ^2^ = 58.19, *df* = 31, RMSEA = 0.07, CFI = 0.95, IFI = 0.95, and TLI = 0.92. Thus, the modified model was ultimately adopted. The results of structural equation modeling showed a significant positive correlation between handwriting fluency and spelling accuracy in fourth grade, with a correlation coefficient of 0.23. There was no significant correlation between handwriting fluency and spelling accuracy in fifth grade. Moreover, handwriting fluency at T2 predicted subsequent spelling accuracy at T3, and spelling accuracy at T2 predicted subsequent handwriting fluency at T3.

## Discussion

This 2-year longitudinal study assessed the bidirectional relationship between handwriting fluency and spelling accuracy. These findings, extending the research of Lam and McBride (2018), confirmed a bidirectional predictive relationship between handwriting fluency and spelling accuracy in upper grade primary school students, even after considering well-established cognitive measures, including non-verbal intelligence, phonological awareness, and rapid automatized naming. However, inconsistent with previous research (Bosga-Stork et al., 2016), this study indicated that handwriting fluency and spelling accuracy in Chinese are still interdependent in the upper grades of primary school, and provided further clarification regarding the extent to which the two predict and facilitate each other.

### Effects of Handwriting Fluency on Spelling Accuracy

Supporting the hypothesis, the current study showed that handwriting fluency in fourth grade significantly predicted subsequent spelling accuracy in fifth grade, which is consistent with previous findings (McBride-Chang et al., 2011; Lam and McBride, 2018). This suggested that previous proficient handwriting fluency could improve later spelling accuracy for both Chinese and alphabetic characters, even after third grade. This could be due to a few possible reasons, discussed below.

First, handwriting improves visual-motor integration, which further develops spelling accuracy. Frith (1982) argued that some children's poor spelling may be the result of a lack of attention to details for letter-by-letter sequencing. Handwriting uses a visual-motor integration process involving perceiving a visual form and responding with hand movements (Lai and Leung, 2012). This facilitates the formulation of children's strategies for analyzing and reproducing different types of Chinese characters, and allows children to mentally code and store characters in a systematic way. Additionally, it could help children develop and shape their own corresponding motor programs, which could achieve the effect of memory consolidation by practice. Practicing handwriting-based movements could help children form basic sensory impressions about the structure type and stroke order of Chinese characters. In this process, the integration of vision and movement is constantly strengthened, and the stroke order is gradually stored in the motor program. When spelling words, subsequent stroke trends are already preformed in the mental lexicon with the help of kinesthetic cues, ensuring the output of correct character for children with consistent strokes. Thus, it is possible for children to improve their spelling accuracy by maintaining consistent handwriting. This is also applicable for upper grade primary school students who are still learning vocabulary.

Second, as was shown in the research of Yan et al. (2012), handwriting may refine word processes, increase orthographic depth, and make meaning representation of characters more precise. A prior study (Cheng-Lai et al., 2013) demonstrated that the lexical knowledge of Chinese characters plays an important role in individual differences in word dictation performance. Therefore, spelling accuracy is, to some extent, dependent on existing knowledge of vocabulary. Spelling accuracy can be achieved with the acquisition of a reasonably large vocabulary and the ability to use familiar words fluently. Handwriting practice, as one of the vital and most common ways to learn Chinese characters, would contribute to becoming familiar with new words for children. Once children have mastered the corresponding Chinese characters through handwriting practice, they should no longer need to rely on phonology, orthography, or morphology to construct information, and will be able to directly and accurately retrieve spelling information from long-term memory (Yan et al., 2012). Thus, early handwriting fluency does affect the development of later spelling accuracy.

Third, the findings of this study supported the hypothesis made regarding automatic information processing (LaBerge and Samuels, 1974), which posits that repeated practice will increase blocks of individual information processing, so as to improve accuracy. The current study extends this view to handwriting, indicating that handwriting practice is not simply a mechanical activity, but can promote the development of spelling accuracy and provide empirical evidence for practical writing teaching. Handwriting fluency could help children free up cognitive and attention resources for more effective information processing (Berninger, 1999). Thus, the meaning of words can be automatically retrieved through continuous repetition (LaBerge and Samuels, 1974). Based on the advantages provided by handwriting fluency, the orthographic structure of characters corresponding to meaning can be retrieved more quickly, and the correct character can be produced more precisely, which contributes to the improvement of spelling accuracy, to varying degrees (Limpo et al., 2018). Hence, handwriting fluency could help to increase children's spelling accuracy.

### Effects of Spelling Accuracy on Handwriting Fluency

The current study suggested that previous spelling accuracy did predict the development of later handwriting fluency, which was consistent with previous research (Martlew, 1992; Sumner et al., 2014). These findings provide empirical support for the lexical quality hypothesis (Perfetti and Hart, 2002), which holds that high quality vocabulary representations can promote the rapid access of vocabulary. Better lexical representations are that individuals have a comprehensive grasp of vocabulary, in which the three dimensions (i.e., phonology, orthography, and semantics) of vocabulary are highly integrated in the mental lexicon. With an increasing frequency of the use of high-quality words, the automatic representation and access paths of these words have already been formed, which correspondingly accelerates the access speed of target words, and guides individuals to write quickly and effectively. Hence, spelling accuracy might support the development of subsequent handwriting fluency.

In contrast to previous English-language studies (Martlew, 1992; Sumner et al., 2014), in which the effect of spelling accuracy on handwriting fluency was mostly seen in children with dyslexia, the current study also found this effect in children without dyslexia. One possible reason is that Chinese is square script and morphosyllabic character, which has a writing system profoundly different from alphabetic language systems (Shu et al., 2003). With Chinese spelling, there are many radicals, and once children are aware of the purpose of morphemes and master these complex radicals precisely and skillfully, they can more accurately identify the orthography in their mental lexicons, therefore, more quickly selecting the correct words during dictation (Packard et al., 2006; Lam and McBride, 2018), even to the point of automatic processing, and their handwriting speed will increase correspondingly (Casalis et al., 2011). Children who develop sensitivity to radicals in the early stages may be more inclined to process radicals as a whole, which creates larger blocks with more information in their brains and reduces the cognitive load, even improves handwriting fluency later. However, if these characters are not learned accurately, it is very likely that confusion will occur in a child's memory, and the child will need more time to select character patterns when hearing the pronunciation during dictation. Stroke combination would then take up more cognitive resources, causing a distinct decrease in handwriting speed. For children who have not accumulated sensitivity to orthographic rules in the early stages, their processing efficiency of words is low. Due to the competition of cognitive resources such as attention and memory load, this fluent handwriting is difficult to maintain, so it will continue to affect their handwriting fluency in the later stage. This could also explain why a continued struggle with spelling accuracy in early childhood is more likely to lead to delayed handwriting output, which is detrimental to children's literacy development (Rønneberg and Torrance, 2017). Thus, early spelling accuracy predicts the development of subsequent handwriting fluency.

### Psychoeducational Implications and Limitations

The findings of the current study are significant for correctly understanding children's handwriting and spelling skills. Previous studies have shown that consistent spelling practice is needed in the first 4 years of primary school (Medwell and Wray, 2010). Handwriting is the best practice for promoting the development of spelling skills (Graham, 2010) and the primary method for cultivating children's literacy (Wu et al., 1999). In terms of pedagogical practice, handwriting practice is necessary, in line with the development of writing, and this also supports the rationale of teachers' requirements for repeated handwriting practice when students are learning to write. When learning new words, it is necessary for children to become familiar with their structure through handwriting to improve the accurate retrieval of these words from memory. However, it is important to stress that these findings should not be misinterpreted as support for asking children to write words too mechanically, which can reduce children's interest in writing. Further, given that the present study has shown the importance of spelling accuracy in children's handwriting fluency, we suggest that during the teaching of spelling, teachers should explain the functions of strokes and radicals of words in detail, so as to help children learn meaningfully. On the basis of mastering the spelling skills of basic radicals and correct glyph structure, children can spell words correctly. Without struggle of how to spell, children can write more words fluently in limited time. On the one hand, it prevents the handwriting difficulties effectively, and on the other hand, it saves more cognitive resources to higher-order cognitive processing, such as logical organization and composition writing.

Despite these findings, some limitations of the current study should be discussed. First, the population was relatively small. In future studies, larger samples could be used to verify these findings. Second, only students in upper primary school grades were followed in the current study. Considering that early grade levels are the critical period for the development of children's literacy, it is necessary to conduct a comprehensive follow-up study to explore the developmental characteristics of handwriting and spelling throughout primary school.

## Data Availability Statement

The datasets generated for this study are available on request to the corresponding author.

## Ethics Statement

The studies involving human participants were reviewed and approved by the Research Ethics Committee of Beijing Normal University. Written informed consent to participate in this study was provided by the participants' legal guardian/next of kin.

## Author Contributions

LL and XW: conception and design of the study. YD, LL, and XW: acquisition, analysis, and interpretation of data. YD and LL: drafting the work and revising it critically for important intellectual content.

### Conflict of Interest

The authors declare that the research was conducted in the absence of any commercial or financial relationships that could be construed as a potential conflict of interest.
