# Supplementary material for: The Reciprocal Relationship Between Handwriting Fluency and Spelling Accuracy in Chinese: A Longitudinal Study
Source: Front Psychol. 2020 Apr 15;11:620. doi: 10.3389/fpsyg.2020.00620 (PMC7174682; doi:10.3389/fpsyg.2020.00620)
Supplement: Supplementary file 1 [file Data_Sheet_1.docx]

**Appendix:**

1、Digit copying fluency task

指导语：请你阅读“一二三四五六七八九十”，阅读后，请又快又准地抄写在下面横线上，抄写完第一遍后请接着抄第二遍、第三遍----，直到我喊停为止。（主试记录1分钟时间）

Instruction: Please read “一二三四五六七八九十(one two three four five six seven eight nine ten)”. After reading, please copy quickly and accurately in the horizontal line below. After copying the first time, please keep copying until I say stop. (The experimenter needs to time one minute)

第一题：一二三四五六七八九十

2、Sentence copying fluency task

指导语：请你阅读“敏捷的棕狐狸跳越懒狗”，阅读后，请又快又准地把抄写在下面横线上，抄写完第一遍后请接着抄第二遍、第三遍----，直到我喊停为止。（主试记录1分钟时间）

Instruction: Please read “敏捷的棕狐狸跳越懒狗 (The quick brown fox jumps over the lazy dog)”. After reading, please copy quickly and accurately in the horizontal line below. After copying the first time, please keep copying until I say stop. (The experimenter needs to time one minute)

第二题：敏捷的棕狐狸跳越懒狗

3、Easy items dictation task

| 四年级 Grade 4 | | |
| --- | --- | --- |
| 序号num | 汉字character | 词语 words |
| 1 | 每 (ever) | 每天 (everyday) |
| 2 | 病 (disease) | 生病 (fall ill) |
| 3 | 表 (table) | 手表 (watch) |
| 4 | 伟 (great) | 伟大 (greatness) |
| 5 | 航 (sail) | 航空 (aviation) |
| 6 | 读 (read) | 读书 (read books) |
| 7 | 怜 (pity) | 可怜 (pitiful) |
| 8 | 闷 (bored) | 闷热 (stuffiness) |
| 9 | 尊 (respect) | 尊敬 (respect) |
| 10 | 详 (detailed) | 详细 (in detail) |
| 11 | 牵 (lead) | 牵手 (hold hands) |
| 12 | 漫 (overflow) | 漫画 (comic) |

| 五年级 Grade 5 | | |
| --- | --- | --- |
| 序号num | 汉字character | 词语 words |
| 1 | 译 (translate) | 翻译 (translation) |
| 2 | 驶 (drive) | 驾驶 (drive) |
| 3 | 忍 (endure) | 忍受 (endure) |
| 4 | 刮 (scratch) | 刮风 (Windy) |
| 5 | 拥 (own) | 拥抱 (embrace) |
| 6 | 饼 (pie) | 饼干 (biscuit) |
| 7 | 测 (measure) | 测试 (test) |
| 8 | 误 (error) | 错误 (error) |
| 9 | 忧 (worry) | 忧伤 (unhappy) |
| 10 | 训 (train) | 教训 (lesson) |
| 11 | 谜 (mystery) | 谜语 (riddle) |
| 12 | 倍 (times) | 加倍 (double) |

4、Difficult items dictation task

| 四年级 Grade 4 | | |
| --- | --- | --- |
| 序号num | 汉字character | 词语 words |
| 1 | 译 (translate) | 翻译 (translation) |
| 2 | 驶 (drive) | 驾驶 (drive) |
| 3 | 忍 (endure) | 忍受 (endure) |
| 4 | 刮 (scratch) | 刮风 (Windy) |
| 5 | 拥 (own) | 拥抱 (embrace) |
| 6 | 饼 (pie) | 饼干 (biscuit) |
| 7 | 测 (measure) | 测试 (test) |
| 8 | 误 (error) | 错误 (error) |
| 9 | 忧 (worry) | 忧伤 (unhappy) |
| 10 | 训 (train) | 教训 (lesson) |
| 11 | 谜 (mystery) | 谜语 (riddle) |
| 12 | 倍 (times) | 加倍 (double) |

| 五年级 Grade 5 | | |
| --- | --- | --- |
| 序号num | 汉字character | 词语 words |
| 1 | 勉 (strive) | 勉强 (reluctant) |
| 2 | 谎 (lie) | 说谎 (tell a lie) |
| 3 | 剧 (drama) | 剧场 (theater) |
| 4 | 辣 (peppery) | 辣椒 (chili) |
| 5 | 豫 (prepare) | 犹豫 (hesitate) |
| 6 | 秩 (order) | 秩序 (order) |
| 7 | 梳 (comb) | 梳头 (comb hair) |
| 8 | 购 (buy) | 购物 (shopping) |
| 9 | 捐 (contribute) | 捐款 (donation) |
| 10 | 凑 (collect) | 凑合 (passable) |
| 11 | 赚 (gain) | 赚钱 (make money) |
| 12 | 瓷 (porcelain) | 瓷器 (porcelain) |
